# Supplementary material for: Identification and functional analysis of non-coding regulatory small RNA FenSr3 in Bacillus amyloliquefaciens LPB-18
Source: PeerJ. 2023 May 15;11:e15236. doi: 10.7717/peerj.15236 (PMC10194069; doi:10.7717/peerj.15236)
Supplement: Supplemental Information 4 [file peerj-11-15236-s004.zip › KO/CK-vs-T1_map/map04614.html]

KEGG PATHWAY: Renin-angiotensin system - Reference pathway


|  |  |
| --- | --- |
| **Renin-angiotensin system - Reference pathway** |  |

[
Pathway menu
| Organism menu
| Pathway entry
| Show description
| User data mapping
]

The renin-angiotensin system (RAS) is a peptidergic system with endocrine characteristics regarding to the regulation of the blood pressure and hydro-electrolytic balance. In the classical RAS, the enzyme renin cleaves its substrate angiotensinogen (Agt) forming the decapeptide angiotensin I that is in turn cleaved by angiotensin-converting enzyme (ACE) to produce the angiotensin II (Ang II), a key player of this system. Ang II activates its AT1 receptor (AT1R), the principal receptor that mediates the majority of the known actions of Ang II in the kidney, including vasoconstriction, renal sodium (Na+) reabsorption, and aldosterone secretion, increasing blood pressure and contributing to the development of hypertension. In addition to (ACE)/Ang II/AT1R and AT2R axis, other signaling pathways in the RAS, such as ACE2/angiotensin-(1-7)/Mas and Ang IV/IRAP, and other active peptide of the RAS, with physiological relevance as Ang III, Ang A and alamandine, are now widely recognized.

|  |  |  |
| --- | --- | --- |
| Reference pathway | 184% 150% 122% 100% 82% 67% 55% | 图片下载 |
